# Switching and loss of cellular cytokine producing capacity characterize *in vivo* viral infection and malignant transformation in human T- lymphotropic virus type 1 infection

**DOI:** 10.1371/journal.ppat.1006861

**Published:** 2018-02-14

**Authors:** Huseini Kagdi, Maria Antonietta Demontis, Juan Carlos Ramos, Graham P. Taylor

**Affiliations:** 1 Section of Virology, Department of Medicine, Imperial College London, London, United Kingdom; 2 Department of Hematology/Oncology, University of Miami School of Medicine, Miami, Florida, United States of America; Vaccine Research Center, UNITED STATES

## Abstract

Adult T-cell leukaemia/lymphoma (ATL) arises from chronic non-malignant human T lymphotropic virus type-1 (HTLV-1) infection which is characterized by high plasma pro-inflammatory cytokines whereas ATL is characterized by high plasma anti-inflammatory (IL-10) concentrations. The poor prognosis of ATL is partly ascribed to disease-associated immune suppression. ATL cells have a CD4+CCR4+CD26-CD7- immunophenotype but infected cells with this immunophenotype (‘ATL-like’ cells) are also present in non-malignant HTLV-1 infection. We hypothesized that ‘ATL-like’ and ATL cells have distinct cytokine producing capacity and a switch in the cytokines produced occurs during leukemogenesis. Seventeen asymptomatic carriers (ACs), 28 patients with HTLV-1-associated myelopathy (HAM) and 28 with ATL were studied. Plasma IL-10 concentration and the absolute frequency of IL-10-producing CD4+ T cells were significantly higher in patients with ATL compared to AC. IL-10-producing ATL cells were significantly more frequent than ‘ATL-like’ cells. The cytokine-producing cells were only a small fraction of ATL cells. Clonality analysis revealed that even in patients with ATL the ATL cells were composed not only of a single dominant clone (putative ATL cells) but also tens of non-dominant infected clones (‘ATL-like’ cells). The frequency of cytokine-producing cells showed a strong inverse correlation with the relative abundance of the largest clone in ATL cells suggesting that the putative ATL cells were cytokine non-producing and that the ‘ATL-like’ cells were the primary cytokine producers. These findings were confirmed by RNAseq with cytokine mRNA expression in ATL cells in patients with ATL (confirmed to be composed of both putative ATL and ‘ATL-like’ cells by TCR analysis) significantly lower compared to ‘ATL-like’ cells in patients with non-malignant HTLV-1 infection (confirmed to be composed of hundreds of non-dominant clones by TCR analysis). A significant inverse correlation between the relative abundance of the largest clone and cytokine mRNA expression was also confirmed. Finally, ‘ATL-like’ cells produced less pro- and more anti-inflammatory cytokines than non ‘ATL-like’ CD4+ cells (which are predominantly HTLV uninfected). In summary, HTLV-1 infection of CD4+ T cells is associated with a change in cytokine producing capacity and dominant malignant clonal growth is associated with loss of cytokine producing capacity. Non-dominant clones with ‘ATL-like’ cells contribute to plasma cytokine profile in patients with non-malignant HTLV-1 infection and are also present in patient with ATL.

## Author summary

Human T-cell lymphotropic virus type-1 (HTLV-1) infection of CD4+ T cells is associated with a change in their cytokine producing capacity and is responsible for the different plasma cytokine profiles in patients with adult T-cell leukaemia/Lymphoma (ATL) and non-malignant HTLV-1 infection. Dominant malignant clonal growth of the infected CD4+ T cells is associated with loss of cytokine producing capacity. ACs, patients with HAM and patients with ATL have a common cytokine cluster with positive correlations between pro- (TNFα and IL-6) and anti- (IL-10) inflammatory cytokines. Plasma IL-10 was higher in the HAM and ATL states compared to AC whilst there was no difference in pro-inflammatory cytokines. Patients with HAM have raised plasma concentrations of IFNγ, IL-10 and IL-17 suggesting a complex interaction between these cytokine in HAM which was not seen in ATL. Aggressive ATL is associated with raised plasma concentrations of pro- and anti-inflammatory cytokines compared to indolent ATL. This cytokine profile did not precede or predict aggressive ATL. The ‘ATL-like’ infected cells in ACs and in patients with HAM have lower pro- and higher anti-inflammatory cytokine secretion than non- ‘ATL-like’ cells which are predominantly HTLV-1 uninfected. Putative ATL cells have little or no cytokine producing capacity. ‘ATL-like’ infected cells from non-dominant infected clones were present not only in patients with non-malignant HTLV-1 infection but also ATL. ‘ATL-like’ cells have cytokine producing capacity and contribute to plasma cytokine profile in patients with non-malignant HTLV-1 infection and possibly also in ATL.

## Introduction

Human T- lymphotropic virus type-1 (HTLV-1) is a complex delta retrovirus infecting an estimated 10 million individuals worldwide [1]. In the majority, infection leads to a chronic asymptomatic carrier state (AC) but 2% to 6% develop adult T-cell leukaemia/lymphoma (ATL) and another 3% inflammatory disorders e.g. HTLV-1-associated myelopathy (HAM). The diagnosis of ATL is based on clinical features, morphology (lymphocytes with characteristic ‘flower cell’ morphology), immunophenotyping (CD3+, CD4+, CCR4+, CD25+, CD26- and CD7-) and demonstration of dominant HTLV-1 infected clones [2, 3]. ATL is classified into four subtypes: smouldering, chronic, acute and lymphoma. Smouldering and chronic ATL are suggested to have an indolent course while acute and lymphoma an aggressive course [3]. Survival with chemotherapy is poor [4–7] due to primary chemo-refractory disease, or early relapse or opportunistic infections. [8–11].

Patients with ATL have high plasma concentrations of anti-inflammatory cytokines e.g. interleukin (IL)-10 [12, 13]. IL-10 is secreted by regulatory CD4+ T cells[14]. CD4+ T cells in patients with ATL have been shown to have high IL-10 expression [13, 15]. ATL cells express regulatory T cell-associated markers CD25 and FOXP3 [16–19]. This has led to the assumption that ATL cells are a regulatory T cell counterpart and mediate an immunosuppressive clinical state by secreting IL-10. In contrast HAM is characterized by organ damage due to immune activation, and high concentrations of plasma pro-inflammatory cytokines e.g. interferon γ (IFNγ) [20]. HTLV-1 infected cells secrete IFNγ [21, 22] and directly contribute to the plasma cytokine profile in HAM. These findings suggest a distinct cytokine producing capacity of HTLV-1 infected cells in keeping with clinical state.

ATL arises *de novo* in AC and patients with HTLV-1-associated inflammation such as HAM, hereafter referred to as non-malignant HTLV-1 infection. HTLV-1-infected cells have a CD4+CCR4+CD26- immunophenotype; the loss of CD7 expression by these cells differentiates ATL from non-malignant HTLV-1 infection [23–27]. We and others have shown that ‘ATL-like’ HTLV-1-infected (CD4+CCR4+CD26-CD7-) cells are present in patients with non-malignant HTLV infection. We hypothesize that the cytokine producing capacity of ATL and ‘ATL-like’ cells are distinct from each other and are directly responsible for the respective plasma cytokine profile in ATL and non-malignant HTLV-1 infection, and that the change in cytokine producing capacity reflects malignant transformation from non-malignant HTLV-1 infection to ATL. Pro- and anti-inflammatory cytokine were measured in plasma and cells (intracellular staining and gene expression by mRNA sequencing) in patients with four different HTLV diagnoses: AC; HAM; indolent ATL; aggressive ATL. Clonality analysis of ATL and ‘ATL-like’ cells was performed and the relationship between plasma, cellular cytokine, clonality and clinical state was studied.

## Material and methods

### Patients and cells

The patient cohort is based at the National Centre for Human Retrovirology (NCHR) at St Mary’s Hospital, Paddington, London, UK and the University of Miami School of Medicine, Miami, USA. Diagnosis of HTLV-1 infection, HAM and ATL was made according to World Health Organization criteria.

### Ethics statement

Patients’ samples are collected and stored in a Communicable Diseases Research Tissue Bank approved by the UK National Research Ethics Service (references 09/H0606/106 and 15/SC/0089). Samples at University of Miami School of Medicines are collected under IRB-approved study "Study of Blood, Tissue and Body Fluids of Viral Associated Malignancies" (EPROST No. 20030608). Samples are stored under license in accordance with the Human Tissues Act 2004. Samples were collected prior to any systemic therapy in patient with ATL or immune modulatory treatment in patients with HAM. Baseline demographic and clinical characteristics of the study population are shown in S1 Table.

Peripheral blood mononuclear cells (PBMCs) and plasma were separated from fresh whole blood by density-gradient centrifugation on Histopaque-1077 (Sigma-Aldrich, St Louis, USA). Plasma was stored at -80°C. PBMCs were harvested from the interface, washed in phosphate buffer saline (PBS, Sigma-Aldrich), cryopreserved in 10% dimethyl sulphoxide (Sigma-Aldrich) and 90% heat inactivated foetal calf serum (FCS) (Gibco, Carlsbad, USA), and stored in liquid nitrogen until use.

### Electrochemiluminescence assay

Plasma cytokine concentrations of the following nine cytokines/chemokines were measured using sensitive and specific V_PLEX immunoassays according to the manufacturer’s protocol (Meso Scale Discovery, Gaithersburg, USA): Human pro-inflammatory cytokines: interferon gamma (IFNγ), interleukin (IL)-2, IL-6, IL-7, IL-17α, tumour necrosis factor-alpha (TNFα); anti-inflammatory cytokine: IL-10 and chemokines: macrophage-derived chemokine (MDC) also known as CCL22 and C-X-C motif chemokine 10 (CXCL-10) also known as IFNγ-induced protein 10 (IP-10),.

For samples with detectable concentrations below the limits of quantification missing values were replaced with 99% of the lowest detectable concentration.

### Cellular cytokine assay

Thawed cryopreserved PBMCs were incubated in either complete media (CM) comprising 10% heat inactivated FCS in RPMI with L-glutamine plus 1% Penicillin only, CM with phytohemaggulutinin (PHA, final concentration of 5 μg/mL, Sigma) or CM with 2% cell activation cocktail (containing phorbol 12-myristate 13-acetate (PMA) and ionomycin, (BioLegend, San Diego, USA)) at 37°C, in 5% CO_2_ at a concentration of 10^6^ cells/ 50 μL for 6 hours. Brefeldin A (BioLegend) was added for the last five hours. All washes were done by suspending cells in PBS containing 1% FCS followed by centrifugation at 600g for 5 minutes twice. PBMCs were washed thrice at the end of incubation and stained sequentially with near infrared fixable viability stain followed by flurochrome conjugated monoclonal antibodies against cell surface markers (CD3, CD4, CD7, CD8 and CCR4) for 30 minutes at room temperature (RT). The cells were then fixed using fixation buffer from FoxP3 / Transcription Factor Staining Buffer Set (eBioscience, San Diego, USA). The fixed cells were washed with permeablization buffer followed by incubation with fluorochrome-conjugated monoclonal antibodies against IL-6, IL-10, TNFα and IFNγ for 15 minutes at RT. PBMCs were then washed twice and stored at 4°C in PBS 1% FCS overnight until analysis on Becton Dickinson Fortessa II. A minimum of 20,000 events were recorded for analysis. Compensation was computed using BD FACS diva software using single staining of Comp ebeads (eBioscience) and checked manually. Fluorescence minus one control (antibody cocktail containing all antibodies except one) was used for gating. Data were analysed by Flowjo software.

### Magnetic cell sorting

Magnetic cell sorting was performing by negative selection. Thawed PBMCs were incubated with primary biotinylated antibodies (CD7, CD26, CD8, CD14, CD15, CD16, CD19, CD36, CD 56, CD123, TCR γ/δ, CD235a [Miltenyi Biotec Ltd., United Kingdom and Biolegend, USA]) at 4°C for 10 min and 10^7^ cells/100 μl. All washes were done by suspending cells in 1% BSA (Sigma) followed by centrifugation at 600g for 5 minutes twice. Labelled PBMCs were washed and incubated with 30% dilution of streptavidin conjugated microbeads at a final concentration of 10^7^ cells/100 μL. Microbead-conjugated PBMCs were washed and magnetically sorted twice on LS Columns (Miltenyi Biotec Ltd) according to the manufacturer’s directions. The flow-through containing CD3+CD4+CD7-CD26-cells, of which 99% were CCR4+, was washed twice. Of these cells, 10^4^ cells were used to check for purity by flow cytometry and the remainder were used for HTLV-1 proviral load quantification and clonality analysis.

### HTLV-1 proviral load (PVL) quantification

Genomic DNA was extracted from PBMCs and sorted CD4+ T cell subsets using QIAamp DNA mini kit (Qiagen, Hilden, Germany). The proviral load was determined by real-time PCR as previously described [28].

### Ligation mediated PCR followed by HTS

Clonality analysis was performed using linker-mediated (LM)-PCR, high-throughput sequencing analysis of HTLV-1 integration sites according to the method previously described [29]. Random fragments of 1 μg genomic DNA (100 ng of sample DNA mixed with 900 ng of uninfected DNA from Jurkat cells) were generated by sonication and ligated to custom, partially double-stranded, DNA adaptors. Nested PCR using specific primers was performed to selectively amplify adaptor-ligated DNA fragments abutting the HTLV-1 3’ LTR. The PCR products from each sample had two unique 8bp multiplexing barcodes and were pooled for sequencing. Paired-end 150-base reads were generated on an Illumina MiSeq. The reads were de-multiplexed using the MiSeq reporter. The linker and primer sequences are listed in S2 Table.

The *in silico* analysis was conducted in shell and R environment. Read 1 and Read 2 were aligned to reference human (hg38, UCSC) and HTLV-1 genomes using Bowtie2[30]. The number of unique integration sites (clones) and the relative and absolute abundance of each unique integration site (clonal size) were calculated as previously described [29].

### RNA sequencing

RNA was extracted from MACS-sorted cells using Qiagen Allprep DNA/RNA column-based extraction kits (Qiagen) as per the manufacturer’s instructions. RNA-seq was performed on DNase-treated samples using TruSeq Stranded mRNA Library Prep Kit (Illumina, United States) as per the manufacturer’s instructions. All sorted cells had the highest RNA quality (> 100 ng RNA and RNA quality score ≥ 8). All sequencing was performed using 50 nucleotide paired-end reads on an Illumina HiSeq 4000 instrument at the Imperial biomedical research centre (BRC) genomics facility, London, United Kingdom.

#### Analysis of T-cell receptor clonality

MiXCR software [31] with standard settings was used to identify TCR alpha and beta CDR3-containing reads present in the RNA sequencing data, generating a list of CDR3s (individual clones) and their relative abundances (clonal fraction = reads of individual clone/reads of all clones). Due to allelic exclusion, each T cell clone should only express one beta chain[32], therefore the most abundant beta clonotype (if present, otherwise alpha) was used to define the relative abundance of the clonal T cell in each subject.

#### Mapping and identification of differentially expressed genes

Before read mapping, clean reads were obtained by removing reads that contained adapter or poly-N, and low quality reads from raw data by using Trimmomatic, a flexible read trimming tools (Version 0.36,[33]). At the same time, Q20, Q30, and GC contents of the clean data were calculated using FastQC (Babraham Bioinformatics). All the downstream analyses were based on the high quality clean data. The normalized and differential gene expression was performed using the new Tuxedo pipeline[34]. The clean reads were aligned to the human genome (version: GRCH38) using the HISAT2 program (V2-2.0.1). We applied Stringtie (Version 0.36) and Ballgown algorithms to identify the significantly differentially expressed genes (p<0.05). Cytokine, chemokine and their receptors gene expression was extracted from the differential gene expression data. Hierarchical clustering was performed to generate an overview of the characteristics of the cytokine gene expression profiles, based on values of significantly differentially expressed transcripts.

### Statistics

Statistical analysis was performed using Graphpad Prism software. The significance of difference in continuous variables between multiple patient groups was determined by a Kruskal-Wallis test with Dunn post-test analysis. The significance of difference in continuous variables between two cell subsets was determined using a Wilcoxon signed-rank test. The significance of difference in contingency variables was determined by a chi-squared test. Differences were considered statistically significant if p <0.05. The correlation between two continuous variables was determined by a non-parametric Spearman test. The Spearman correlation was considered significant for p<0.05 and showing a trend if the p value was between 0.05 and 0.1.

A classification tree was constructed to identify the hierarchical organisation of plasma cytokine concentration in ACs, patients with HAM and patients with ATL. The classification tree was produced using a Recursive Partitioning and Regression Trees (RPART) analysis in R.

The network analysis was performed using NodeXL. In the analysis, absolute CD3+, CD4+ and CD8+ cell counts and PBMC PVL were used as cellular variables, and plasma cytokine concentrations were used as cytokine variables. The cellular and cytokine variables were used as edges of the network while the Spearman correlates between nodes were used as vertices. Vertices with at least a significant trend on Spearman correlation (p <0.1) were included. The layout was performed using Harel-Koren Fast multiscale.

## Results

### Plasma cytokine profile in non-malignant HTLV-1 infection and ATL

**Fig 1:**
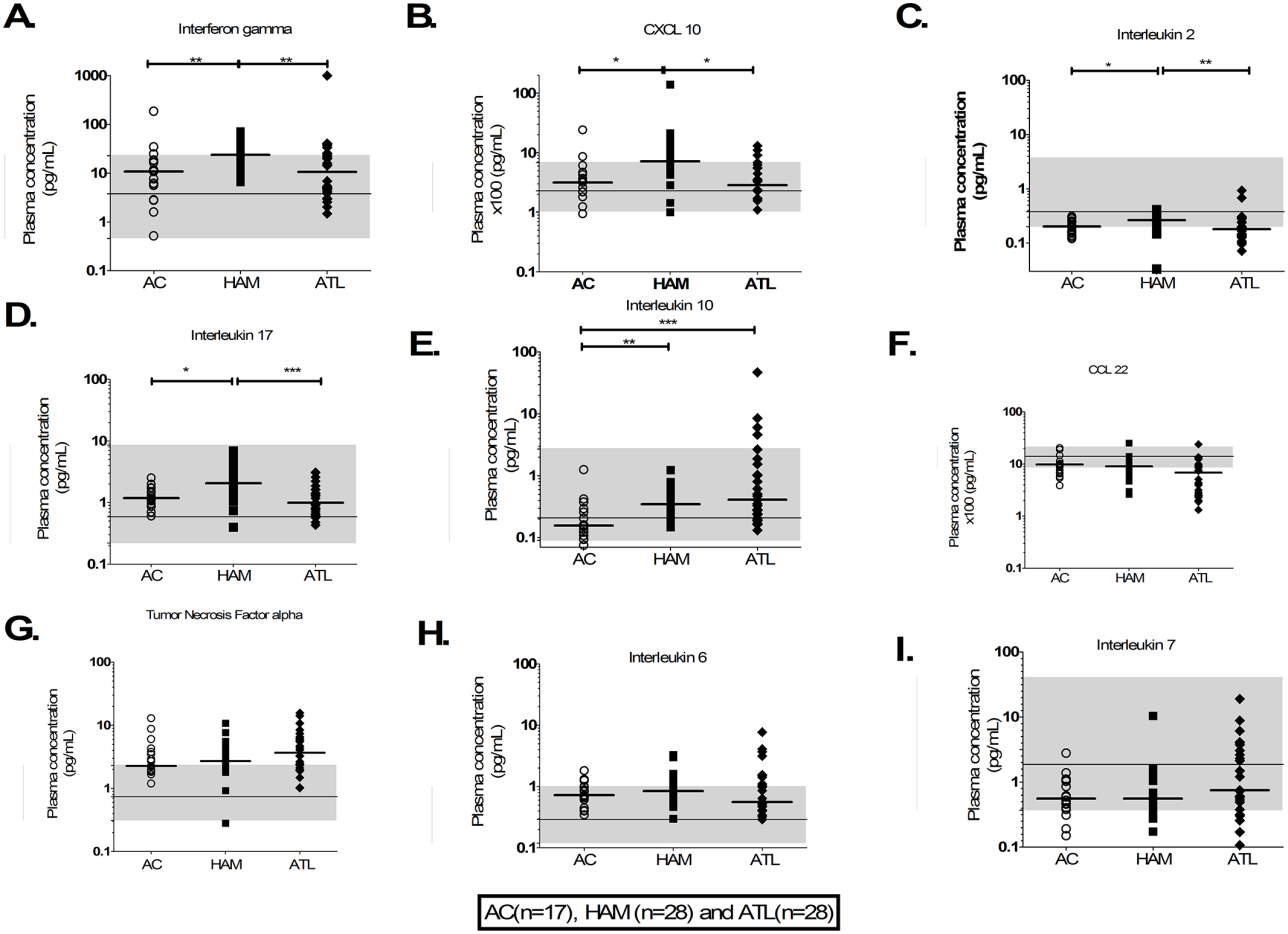
Plasma cytokine concentration in non-ATL HTLV-1 infection and ATL. A-I) Aligned column plots of plasma cytokine/chemokine concentrations in patients with non-ATL HTLV-1 infection and ATL. The bar represents median values. The continuous line and shaded area shows manufacturer supplied median and range in healthy individuals. Statistical analysis: Kruskal-Wallis test with Dunn post-test, 95% confidence interval. * denotes p<0.05, ** denotes p<0.01, *** denotes p<0.001.

The relative and absolute frequencies of CD3+, CD4+ T cells and HTLV-1 PVL in PBMCs were significantly higher in patients with ATL compared to ACs or patients with HAM (Table 1). Patients with HAM had significantly higher plasma concentrations of IFNγ, CXCL10, IL-2 and IL-17 (pro-inflammatory cytokines) compared to ACs and patients with ATL (Fig 1A–1D). Patients with ATL and patients with HAM had significantly higher plasma concentrations of the anti-inflammatory cytokine IL-10 compared to ACs (Fig 1E). There was no significant difference in plasma concentrations of IL-6, IL-7, CCL22 and TNFα between the three patient groups (Fig 1F–1I). The median plasma concentrations of IL-6 and TNFα in all three patient groups were near the upper limit of the manufacturer’s normal human range while those of CCL22, IL-2 and IL-7 were near the lower limit.

**Table 1:** T-cell subsets and proviral load in AC, HAM and ATL.

Patient group	Parameter	CD3		CD4		CD8		PVL (copies per 100 PBMCS)
		Absolute count (10^6/uL)	Relative count (% lymphocytes)	Absolute count (10^6/uL)	Relative count (% lymphocytes)	Absolute count (10^6/uL)	Relative count (% lymphocytes)	
AC (n = 17)	Minimum	881	61.4	539	30	194	12	<0.1
	25% Percentile	1139	67	860	45	289	15	0.7
	Median	1454	73	989	53	385	19	2.8
	75% Percentile	1852	79	1260	58	518	27	18.9
	Maximum	2970	87	1563	66	1470	42	27.9
	Mean	1458	73	1049	51	461	22	9.4
	Std. Deviation	631	8	301	9	360	10	10.4
	Std. Error	158	2	78	2	90	2	2.5
HAM (n = 28)	Minimum	723	59	264	22	232	17	0.4
	25% Percentile	1112	74	678	44	342	22	4.4
	Median	1347	79	864	50	508	25	6.9
	75% Percentile	2057	84	1301	55	855	36	12.6
	Maximum	3768	95	2368	60	1495	73	28.8
	Mean	1644	78	1012	48	633	30	9.0
	Std. Deviation	789	9	522	10	371	13	7.2
	Std. Error	147	2	97	2	69	2	1.3
ATL (n = 28)	Minimum	574	38	334	22	160	1	2.0
	25% Percentile	2345	81	1454	58	287	3	20.3
	Median	6494	93	5499	83	366	8	29.8
	75% Percentile	11868	95	10600	91	531	16	64.6
	Maximum	91000	99	11612	94	1252	31	276.2
	Mean	14534	85	5695	73	446	10	54.3
	Std. Deviation	24674	16	4343	22	263	9	59.9
	Std. Error	5384	4	1024	5	62	2	11.8
Significance of difference?	AC vs HAM	Ns	Ns	ns	ns	ns	ns	ns
	AC vs ATL	***	***	*	***	ns	*	***
	HAM vs ATL	***	***	***	***	ns	***	***

* denotes p<0.05,

*** denotes p<0.001, ns denotes not significant

AC denotes asymptomatic carriers, HAM denotes HTLV-1-associated myelopathy, ATL denotes adult T-cell leukaemia/lymphoma

**Fig 2:**
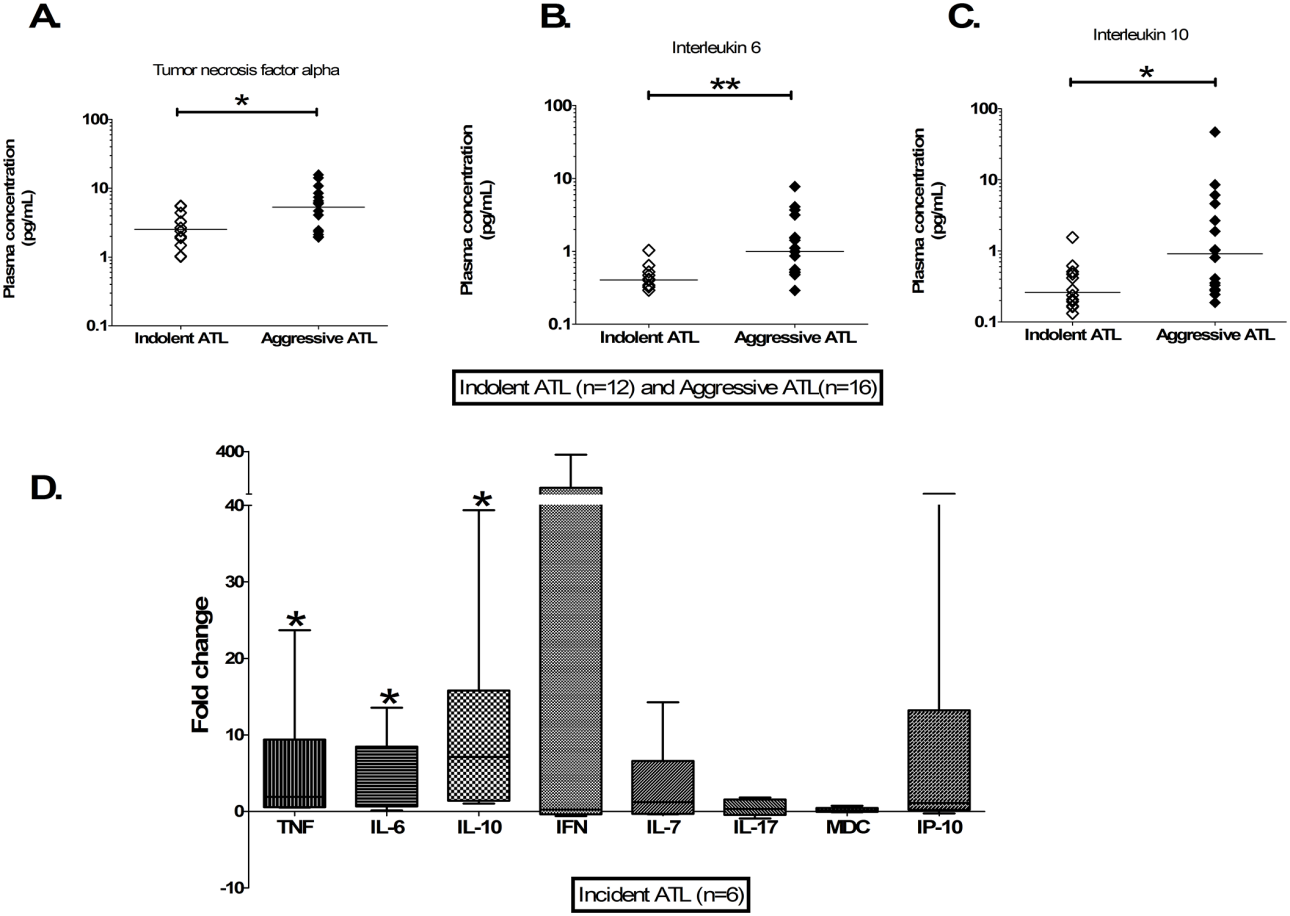
Plasma cytokine concentrations in ATL. A-C) Aligned column plots of plasma cytokine/chemokine concentrations in indolent and aggressive ATL. D) Box plots of fold change in six patients with aggressive ATL at diagnosis and at 3–12 months pre-diagnosis. The bar represents median values. Statistical analysis: Kruskal-Wallis test with Dunn post-test, 95% confidence interval and Wilcoxon signed rank test. * denotes p<0.05, ** denotes p<0.01, *** denotes p<0.001.

Patients with aggressive ATL had significantly higher TNFα, IL-6 and IL-10 plasma concentrations than those with indolent ATL (Fig 2A–2C). There was no significant difference in plasma concentrations of IFNγ, CXCL10, CCL22, IL-2 and IL-7 between ATL subtypes as shown in S1 Fig.

The plasma chemokine and cytokine concentrations, of four patients with HAM who developed *de novo* aggressive ATL and two patients with indolent ATL who progressed to aggressive ATL, were measured at aggressive ATL diagnosis and at 3–12 months earlier. Plasma TNFα, IL-6 and IL-10 concentrations were significantly higher at diagnosis, with median increases of 1.9-fold; 3.7-fold and 7.1-fold respectively (Fig 2D). There was a large variance in changes of plasma IFNγ concentrations.

In summary, patients with HAM have the highest concentrations of pro-inflammatory cytokine and ACs had the lowest IL-10 plasma concentrations. Patients with aggressive ATL had higher plasma concentrations of TNFα, IL-6 and IL-10 compared to patients with indolent ATL. These higher plasma concentrations did not precede malignant progression.

### Network and classification tree analysis of immune profile in non-malignant HTLV-1 infection and ATL

**Fig 3:**
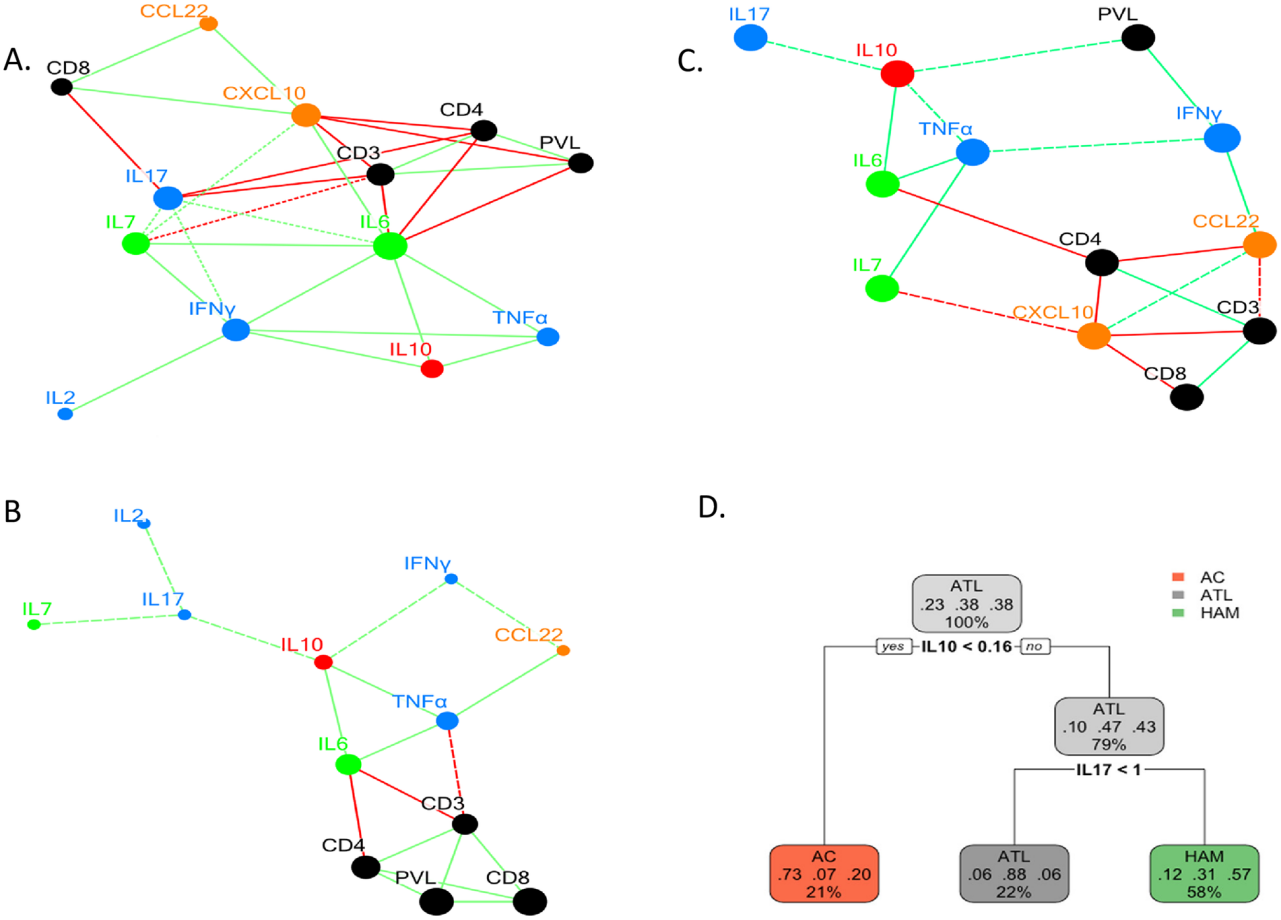
Network and classification tree analysis of plasma cytokine concentration in non-ATL HTLV-1 infection and ATL. Network analysis of absolute T-cell subset count, HTLV-1 PVL and plasma cytokine concentration using at least significant Spearman correlation trends is shown for patients with ATL (A), HAM (B) and AC (C). The green and red lines denote positive and negative correlations respectively. The continuous and broken line denote statistically significant and trend correlations. The prune classification tree to classify diagnosis of AC, HAM and ATL on the basis of IL-10 and IL-17 concentration is shown in figure D. The percentage shows the distribution of all patients into different arms whilst the three decimal numbers shown specificity of each classification for diagnosis of AC, ATL and HAM respectively.

Network analysis was performed to better understand the interaction between cellular and plasma immune markers. The absolute frequency of CD3+, CD4+ T cells and PVL were significantly positively correlated with each other in patients with HAM or ATL (Fig 3A and 3B). There were also significant positive correlations between the plasma concentrations of TNFα, IL-6 and IL-10 in all three diagnostic groups (Fig 3A–3C). The plasma concentration of IL-10 correlated significantly with IFNγ in patients with ATL and to a lesser extent with both IFNγ and IL-17 in patients with HAM.

A classification tree analysis was performed on plasma cytokine concentrations to identify the cytokine profile which best differentiated AC, HAM and ATL states. A plasma IL-10 concentration < 0.16 pg/mL identified AC with a 64.7% sensitivity and 73% specificity as shown in Fig 3D. A plasma IL-17 concentration <1 pg/mL or if the IL-17 concentration was >1 pg/mL an IL-10 concentration of >0.8 pg/mL identified ATL with 78.5% sensitivity and 85% specificity whilst an IL-17 concentration of ≥ 1 pg/mL with an IL-10 concentration between 0.16 and 0.8 pg/mL identified HAM with 82.1% sensitivity and a specificity of 71.8%.

In summary, a positive correlation between the plasma concentrations of specified pro- and anti-inflammatory cytokines was present in all three HTLV-1 patient groups. Together, the plasma concentrations of IL-10 and IL-17 discriminated between the clinical states associated with HTLV-1 infection.

### Cellular cytokine studies in non-malignant HTLV-1 infection and ATL

To identify the cellular source of the cytokines identified in plasma, the cytokine producing capacity of monocytes, CD4+ and CD8+ T cells was studied. Intracellular cytokine staining for TNFα, IFNγ, IL-6 and IL-10 was performed in 10 ACs, 11 patients with HAM and 10 with ATL (four with indolent and six with aggressive ATL). The gating strategy to detect cytokine producing cells is shown in S2 Fig. The relative and absolute frequencies of cells secreting each cytokine are shown as percentages and cell count per litre.

**Fig 4:**
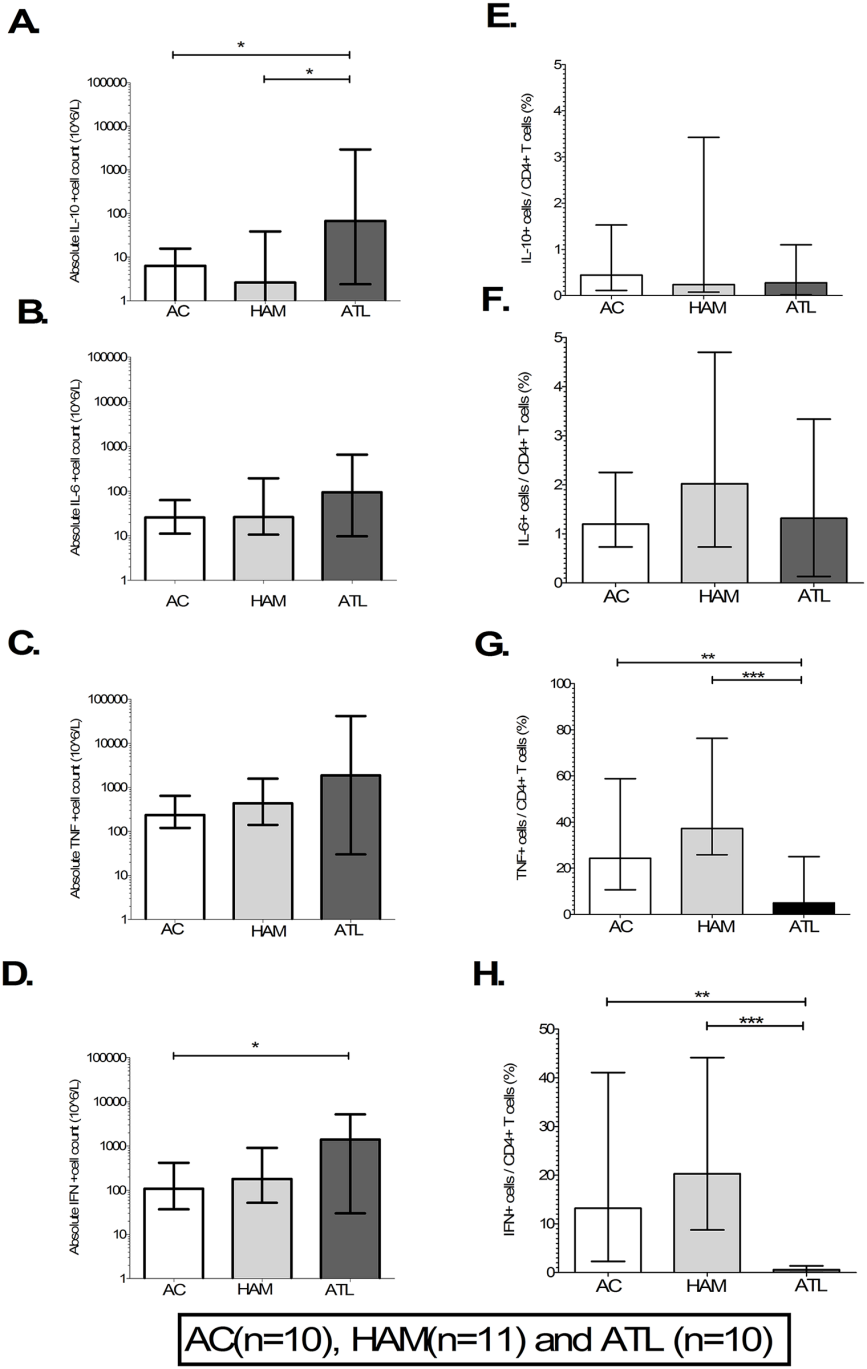
CD4+T-cell cytokine producing profile in non-ATL HTLV-1 infection and ATL. A-H) Bar column plots showing absolute and relative frequency of CD4+T cells in asymptomatic carriers (AC), patients with HTLV-1 associated myelopathy (HAM) and adult T-cell leukaemia/lymphoma (ATL). The bar represents mean values and error bar the standard deviation. Statistical analysis: Kruskal-Wallis test with Dunn post-test, 95% confidence interval and Wilcoxon signed rank test. * denotes p<0.05, ** denotes p<0.01, *** denotes p<0.001.

Although there was no difference in the relative frequency of IL-10+ CD4+ cells (Fig 4E) their absolute frequency was higher in patients with ATL compared to AC and HAM (Fig 4A). The relative and absolute frequency of IL-6+ CD4+ cells did not differ by disease state (Fig 4B–4F). The absolute frequencies of TNFα+ CD4+ T-cells were the same in each group (Fig 4C) but TNF+ cells made up a significantly lower percentage of all CD4+ T cells in patients with ATL compared to non-malignant HTLV-1 infection (Fig 4G). Finally, although the absolute frequency of CD4+ T-cells secreting IFNγ was increased in ATL (Fig 4D), the relative frequency of these cells was significantly lower in patients with ATL than in ACs and patients with HAM (Fig 4H). In patients with ATL the median relative frequencies of TNFα, IFNγ, IL-6 and IL-10 secreting CD4+ cells were 5%, 1.7%, 0.6% and 0.3% respectively. The frequencies of TNFα, IFNγ, IL-6 and IL-10-secreting CD8+ cells and monocytes (except IFNγ, which is not secreted by monocytes) did not differ between diagnostic groups as shown in S3 Fig.

In summary, although the absolute frequency of the cytokine-producing CD4+ T-cells was greater in patients with ATL these make up only a minority of their CD4+ T cells suggesting that ATL cells are in general not secreting these cytokines.

### Cytokine producing capacity of ATL and ‘ATL-like’ cells

CD4+ T cells are the dominant reservoir of infected cells in both non-malignant HTLV-1 infection and ATL [23, 35]. The infected cells are derived from thousands of non-dominant clones in non-malignant HTLV-1 infection and from a dominant clone on a polyclonal background of non-dominant clones in ATL [29, 36, 37]. To further characterise the ATL and ‘ATL-like’ cells, their cytokine producing capability was determined. ATL cells have a CD4+CCR4+CD26-CD7- immunophenotype and ‘ATL-like’ cells are present in non-malignant HTLV-1 infection. The CD4+CCR4+CD7- immunophenotype was used to study the cytokine producing capacity of ATL cells as >99% of CD4+CCR4+CD7- cells were also CD26-.

**Fig 5:**
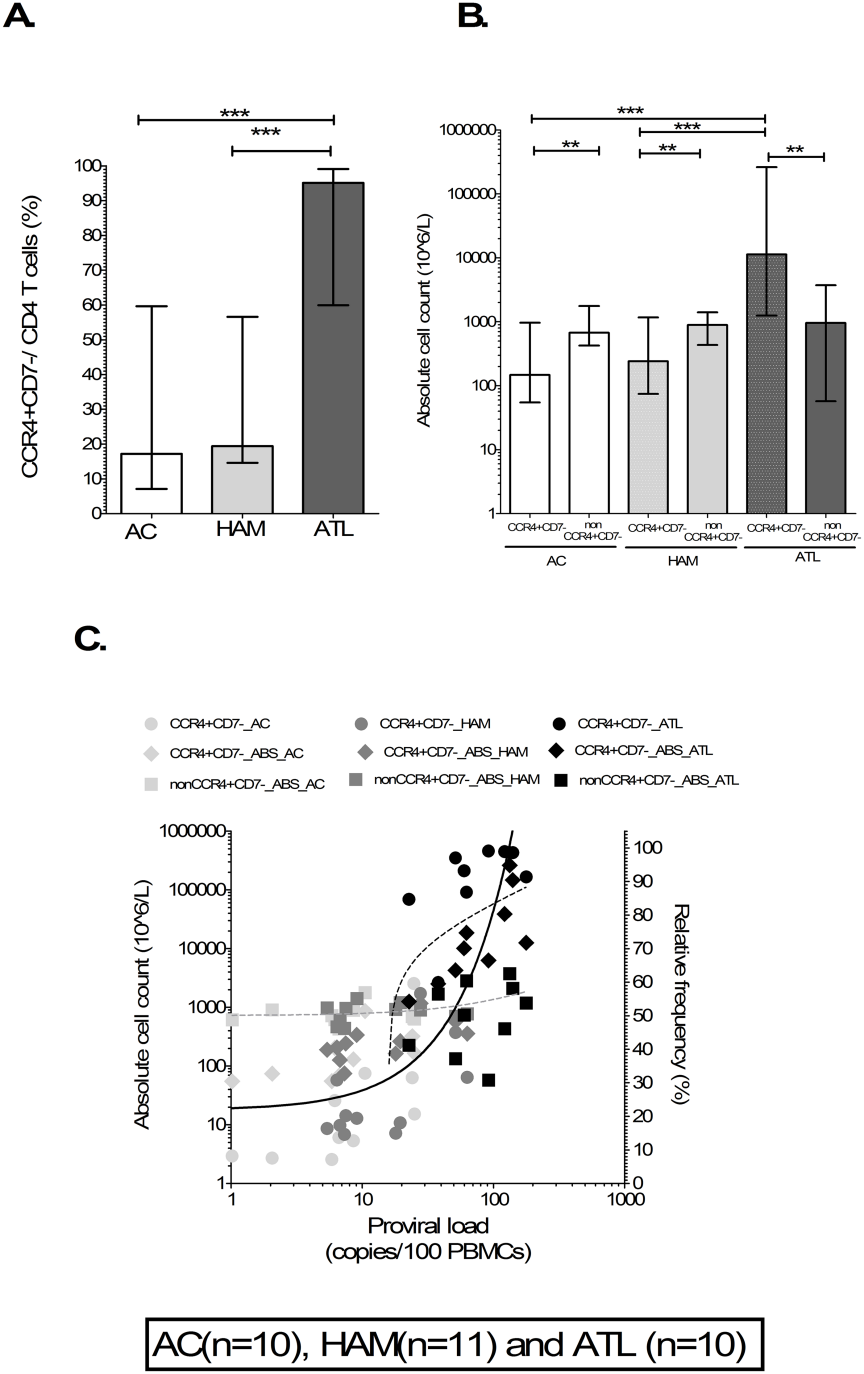
CD4+CCR4+CD7- T cells as marker of ‘ATL-like’ infected and ATL cells. Bar column plots showing the absolute frequency and relative of CD4+CCR4+CD7- (A & B) and non CCR4+CD7- CD4+T cells in AC, patients with HAM and ATL showed highly significant increased frequency of CD4+CCR4+CD7- T cells in patients with ATL compared to AC and HAM. The bar represents median values. Statistical analysis: Kruskal-Wallis test with Dunn post-test, 95% confidence interval and Wilcoxon signed rank test. C) XY scatters plots showing significant positive correlation of PVL with relative and absolute frequency of CD4+CCR4+CD7- T cells. The line represents linear regression line with rho and R for Spearman and linear regression correlate respectively. * denotes p<0.05, ** denotes p<0.01, *** denotes p<0.001.

The relative (Fig 5A) and absolute frequencies (Fig 5B) of CD4+CCR4+CD7- T cells were significantly higher in patients with ATL compared to non-malignant HTLV-1 infection whilst there was no difference in the absolute frequency of non-CCR4+CD7- CD4+ T cells (Fig 5B). HTLV-1 PVL significantly and positively correlated with the relative (rho = 0.87, p<0.0001) and absolute (rho = 0.88, p<0.0002) frequencies of CD4+CCR4+CD7- cells but not the absolute frequency of non-CCR4+CD7- CD4+ T cells (rho = 0.20, p = 0.27). This confirms CD4+CCR4+CD7- cells as marker of ATL and ‘ATL-like’ HTLV-1 infected cells in patients with ATL and non-malignant HTLV-1 infection respectively. HTLV-1 infection leads to an absolute increase in ‘ATL-like’ cells in patients with non-malignant HTLV-1 infection. The relative and absolute frequency of ATL cells was actually higher than non-ATL CD4+T cells in patients with ATL but there was no difference between ‘ATL-like’ and non- ‘ATL-like’ CD4+ T cells in non-malignant HTLV-1 infection.

**Fig 6:**
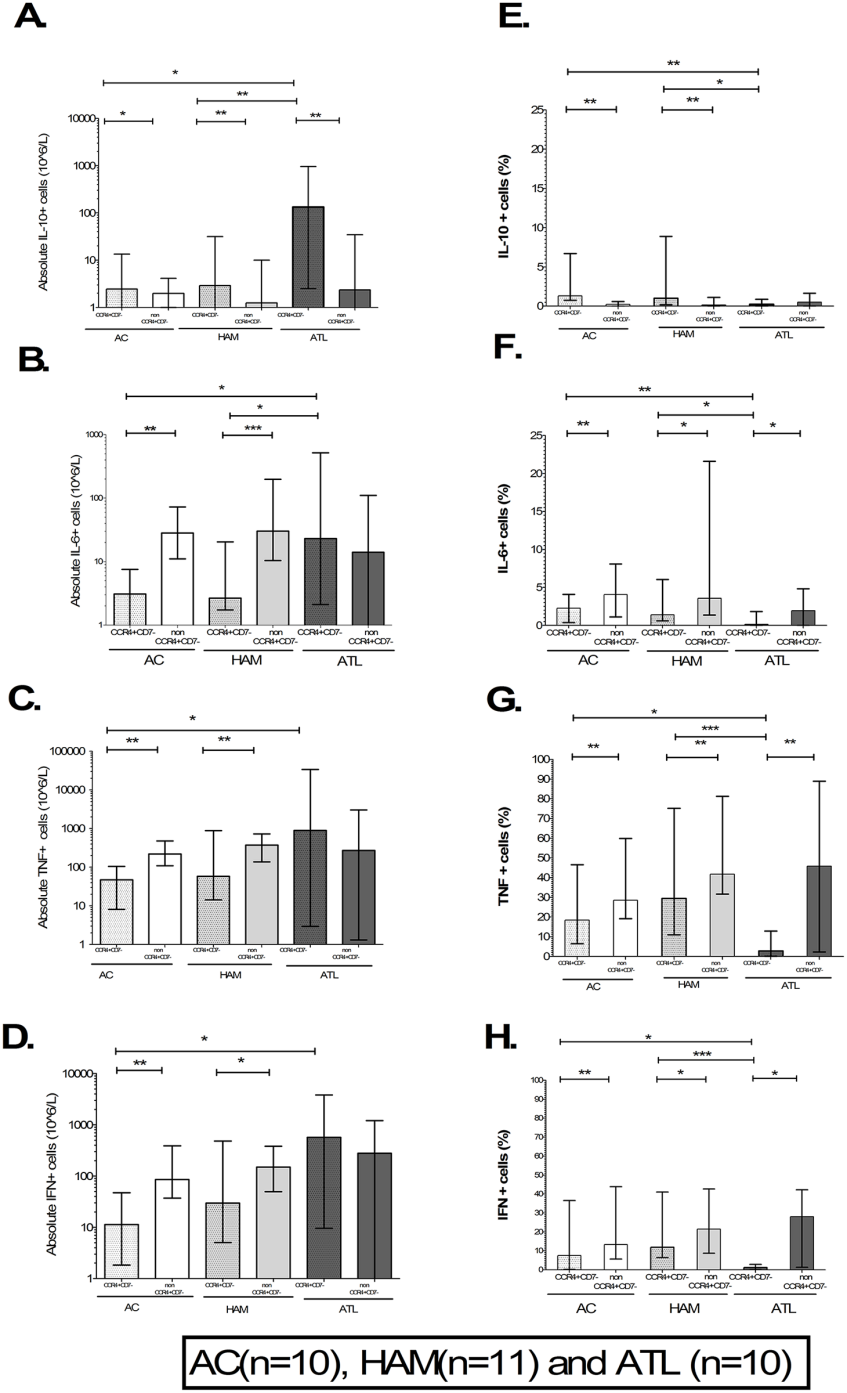
CD4+T-cell subsets cytokine producing profile in non-ATL HTLV-1 infection and ATL. A-H) Bar column plots showing absolute and relative frequency of CCR4+CD7- and non CCR4+CD7- CD4+T-cell subset in asymptomatic carriers (AC), patients with HTLV-1 associated myelopathy (HAM) and adult T-cell leukaemia/lymphoma (ATL). The bar represents mean values and error bar the standard deviation. Statistical analysis: Kruskal-Wallis test with Dunn post-test, 95% confidence interval and Wilcoxon signed rank test. * denotes p<0.05, ** denotes p<0.01, *** denotes p<0.001.

The absolute frequency of IL-10-producing CD4+CCR4+CD7- cells in patients with ATL was significantly higher compared to ACs and HAM (Fig 6A). The relative frequency of IL-10-producing CD4+CCR4+CD7- cells in patients with ATL was lower compared to AC and HAM (Fig 6E). The absolute frequency of IL-6-producing CD4+CCR4+CD7- cells in patients with ATL was higher compared to ACs and patients with HAM (Fig 6B). The relative frequency of IL-6-producing CD4+CCR4+CD7- cells in patients with ATL was low compared to AC and patients with HAM (Fig 6F). The absolute frequency of CD4+CCR4+CD7- T cells producing TNFα (Fig 6C) or IFNγ (Fig 6D) was significantly higher in patients with ATL compared to ACs whilst there was a trend when compared to patients with HAM (p = 0.11 and p = 0.10 respectively). However, the relative frequencies of TNFα and IFNγ producing CD4+CCR4+CD7- cells were significantly lower in patients with ATL compared to ACs and HAM as shown in Fig 6G and 6H. The median relative frequencies of TNFα, IFNγ, IL-6 and IL-10 secreting CD4+CCR4+CD7- T cells in patients with ATL were 3.3%, 1.7%, 0.2% and 0.3% respectively.

The CD4+ T cells which are not CCR4+CD7- are a mixture of predominantly uninfected (82%) and infected cells (18%) [23]. The uninfected non-CCR4+CD7- CD4+T cells are a mix of naïve and memory CD4+T cells. CD4+naïve T cells have been shown to be depleted in HTLV-1 infection. In addition, naïve CD4+T cells produce less cytokine than memory T cells suggesting that the cytokine producing capacity of non-CCR4+CD7- CD4+T cells is mainly derived from uninfected memory T cells. The absolute and relative frequencies of pro- inflammatory cytokine (TNFα, IFNγ and IL-6) producing CCR4+CD7- cells (i.e. ‘ATL-like’ infected cells) was lower than in non-CCR4+CD7- CD4+ T cells (i.e. predominantly HTLV-1 uninfected memory cells, Fig 6F–6H) in AC and patients with HAM. The absolute and relative frequencies of CD4+CCR4+CD7- cells producing the anti-inflammatory cytokine IL-10 was higher compared to non-CCR4+CD7- CD4+T cells in ACs and patients with HAM as shown in Fig 6A–6H. The relative frequency of pro-inflammatory cytokine producing CD4+CCR4+CD7- cells was lower than non-CCR4+CD7- CD4+T cells whilst the absolute frequency of IL-10 secretion was higher in patients with ATL. The absolute and relative frequencies of TNFα, IFNγ, IL-6 and IL-10 secreting non-CCR4+CD7- CD4+cells did not differ significantly between the three clinical groups.

In summary, patients with non-malignant HTLV-1 infection have an increased frequency of CD4+CCR4+CD7- (‘ATL-like’ infected cells) which correlates strongly with PVL. There is a further increase in these cells in patients with ATL. These cells are capable of producing pro- and anti-inflammatory cytokines. However, CD4+CCR4+CD7- cells had lower pro- and higher anti-inflammatory cytokine producing capacity compared to non-CCR4+CD7- CD4+T cells (predominantly uninfected cells) in non-malignant HTLV-1 infection. The absolute frequencies of not only IL-10 cytokine secreting CD4+CCR4+CD7- cells but also TNFα, IFNγ and IL-6 secretors were higher in ATL compared to non-malignant HTLV-1 infection. However, the cytokine producing cells made up only a tiny fraction of CD4+CCR4+CD7- T cells in patients with ATL. This suggests that at some point in the transformation of ‘‘ATL-like’ to ‘ATL’ cells CD4+CCR4+CD7- lose their cytokine producing capacity and that they are not the source of the plasma cytokines observed in ATL. The question remains whether the cytokine-secreting ‘ATL’ cells are a sub-population of the malignant clone or ‘ATL-like’ cells.

### Clonality within CD4+CCR4+CD7- T cells in ATL

Patients with ATL have a putative dominant clone on a polyclonal background. The dominant cell population in ATL is CD4+CCR4+CD7-. In order to determine the likely clonal origin of cytokine secreting CD4+CCR4+CD7- T cells clonality analysis was performed within sorted CD4+CCR4+CD7- T cells in four patients with aggressive ATL. In these patients, the CD4+CCR4+CD7- cells population had a median of 41 clones with the largest clone contributing a median 88% of the HTLV-1 infection burden as shown in Table 2. Thus, CD4+CCR4+CD7- cells in patients with ATL are derived not exclusively from a single dominant clone (putative ATL cells) but also from tens of non-dominant infected clones i.e. ‘ATL-like’ cells. There was a perfect negative correlation (rho = -0.99, p<0.0001) between the relative frequencies of cytokine secreting cells and the relative abundance of the largest clone further supporting the suggestion that ‘ATL-like’ infected cells from non-dominant clones are cytokine producing whilst the cells from the dominant clone secrete little or no cytokines in patients with ATL.

**Table 2:** Clonality and cytokine producing profile of CD4+CCR4+CD7- T cells in patients with ATL.

Patient code	Clinical state	Proviral load (copies per 100 cells)	Total number of HTLV-1 infected clones	Relative abundance of the largest clones (%)	Cells secreting any cytokine (%)
ATL12	Aggressive ATL	178.4	41	82	28
ATL22	Aggressive ATL	165.7	34	94	6
ATL197	Aggressive ATL	179.4	40	89	16
LGL2	Aggressive ATL	229.0	50	86	23
Median	na	178.9	41	88	19

### Inflammatory transcriptome of CD4+CCR4+CD7- cells in patients with non-malignant HTLV-1 infection and ATL

In order to determine the global and confirm the deferential cytokine profile of CD4+CCR4+CD7- cells in patients with non-malignant HTLV-1 infection and ATL, we studied the expression of cytokines, chemokines and their receptors by mRNA sequencing of sorted CD4+CCR4+CD7- T cells in eight patients with non-malignant (four AC and four patients with HAM) and eight with ATL.

#### Clonality within CD4+CCR4+CD7- cells in patients with non-malignant and ATL

Clonality analysis within these sorted cells was performed by extracting T-cell receptor (TCR) alpha and Beta sequences using MiXCR pipeline ([31]).

There was no difference in total and reads aligned to TCR-Beta and TCR-alpha in patients with non-malignant HTLV-1 infection and ATL as shown in Table 3. A median of 0.009% and 0.0046% of all reads were aligned to TCR-Beta sequence in patients with ATL and non-malignant HTLV-1 infection respectively. A significantly larger number clones were detected within ‘ATL-like’ cells in patients with non-malignant (median = 341) HTLV-1 infection compared to ATL (median = 21, range, p = 0.0002) whilst the clonal fraction of the two largest clones was significantly lower in patients with non-malignant (median = 0.07) compared to ATL (median = 0.99, p = 0.0012) as shown in Table 3. Six out of eight patients with ATL had a single clone contributing more than 0.94 of the total by TCR-Beta clonality analysis. One patient with ATL (LFA) had two similar size clones whilst one (LGV) had very low alignment to TCR Beta sequences. Both these patients had a single clone (LFA = 0.92 and LGV = 0.95 respectively) by TCR-alpha clonality analysis. This suggests that the lower clonal fraction by TCR-Beta clonality in these patients is due to bi-allelic TCR-Beta expression and low TCR-Beta mapped reads respectively. In all but one patient with non-malignant infection the two largest clones contributed less than 0.15 of the total clonal fraction. One patient with non-malignant infection (HKU) had two large clones by both TCR-Beta and TCR-alpha clonality analysis. These data confirm that CD4+CCR4+CD7- cells are derived from a large number of non-dominant clones in patients with non-malignant HTLV-1 infection and a single dominant clone on a background of non-dominant in patients with ATL.

**Table 3:** TCR clonality within ‘ATL-like’ cells in patient with non-malignant HTLV-1 infection and ATL.

Patient group	CODE	Total reads (x10^6)	TCR-Beta						TCR-alpha					
			Reads aligned to CDR3	Read aligned to CDR3 per 10^4 total reads	Number of clones	clonal Fraction of largest clone	clonal Fraction of second largest clone	clonal Fraction of two largest clone	Reads aligned to CDR4	Read aligned to CDR3 per 10^4 total reads	Number of clones	clonal Fraction of largest clone	clonal Fraction of second largest clone	clonal Fraction of two largest clone
ATL	ATL12	52	4726	9	16	0.99	0.00	1.00	1533	3	7	0.88	0.11	0.99
	ATL22	72	23775	33	7	1.00	0.00	1.00	2430	3	3	0.99	0.00	0.99
	ATL197	79	8111	10	10	1.00	0.00	1.00	3131	4	9	0.98	0.01	0.99
	LFA	73	9175	13	33	0.55	0.43	0.98	4663	6	19	0.93	0.06	0.99
	LFV	70	3453	5	3	1.00	0.00	1.00	2036	3	4	0.68	0.32	1.00
	LGL	130	14027	11	38	0.95	0.03	0.98	7073	5	37	0.85	0.10	0.95
	LGV	66	35	0	26	0.09	0.09	0.17	2683	4	13	0.95	0.05	1.00
	LHN	70	2399	3	48	0.95	0.01	0.96	1190	2	33	0.68	0.28	0.96
	Median	71	6419	10	21	0.97	0.01	1.00	2557	4	11	0.91	0.08	0.99
non-malignant	HES	50	2322	5	322	0.04	0.03	0.07	907	2	161	0.02	0.02	0.04
	HHL	78	2532	3	290	0.07	0.06	0.13	1779	2	183	0.23	0.03	0.26
	HKQ	9	380	4	72	0.04	0.03	0.07	185	2	39	0.07	0.04	0.11
	HKU	57	2385	4	181	0.40	0.32	0.73	1062	2	112	0.34	0.13	0.47
	TBG	86	5529	6	1130	0.04	0.03	0.06	2221	3	667	0.05	0.03	0.08
	TCD	117	8276	7	2531	0.01	0.01	0.02	4506	4	1750	0.01	0.01	0.02
	TCX	155	11643	8	1385	0.09	0.03	0.12	5702	4	1157	0.06	0.03	0.09
	TDU	91	4123	5	359	0.02	0.02	0.03	2494	3	248	0.02	0.02	0.04
	Median	82	3328	5	340.5	0.04	0.03	0.07	2000	2	216	0.06	0.03	0.09
significant of difference between ATL and non-malignant	p value	0.72	0.38	0.23	0.0002	0.0016	0.1971	0.0012	0.33	0.11	0.0002	0.0009	0.21	0.0009
	Comment	not significant	not significant	not significant	***	**	not significant	**	not significant	not significant	***	***	not significant	***

#### Differential cytokine expression within CD4+CCR4+CD7- cells in patients with non-malignant HTLV-1 infection and ATL

We performed differential inflammatory transcriptome expression (cytokines, chemokines and their receptor mRNA expression) within ‘ATL-like’ cells in patient with non-malignant HTLV-1 infection and ATL. A total of 127 inflammatory transcripts were detected in at least one patient as shown in S3 Table. Seventeen inflammatory transcripts were differentially expressed (p<0.05) in patients with non-malignant infection compared to ATL, out of which ten (CCL5, CXCR3, CXCR4, CXCR5, CCR5, CCR6, IFNG, TNF, ICAM1 and IL-10RA) were expressed ≥ 2-fold higher in patient with non-malignant compared to ATL infection.

**Fig 7:**
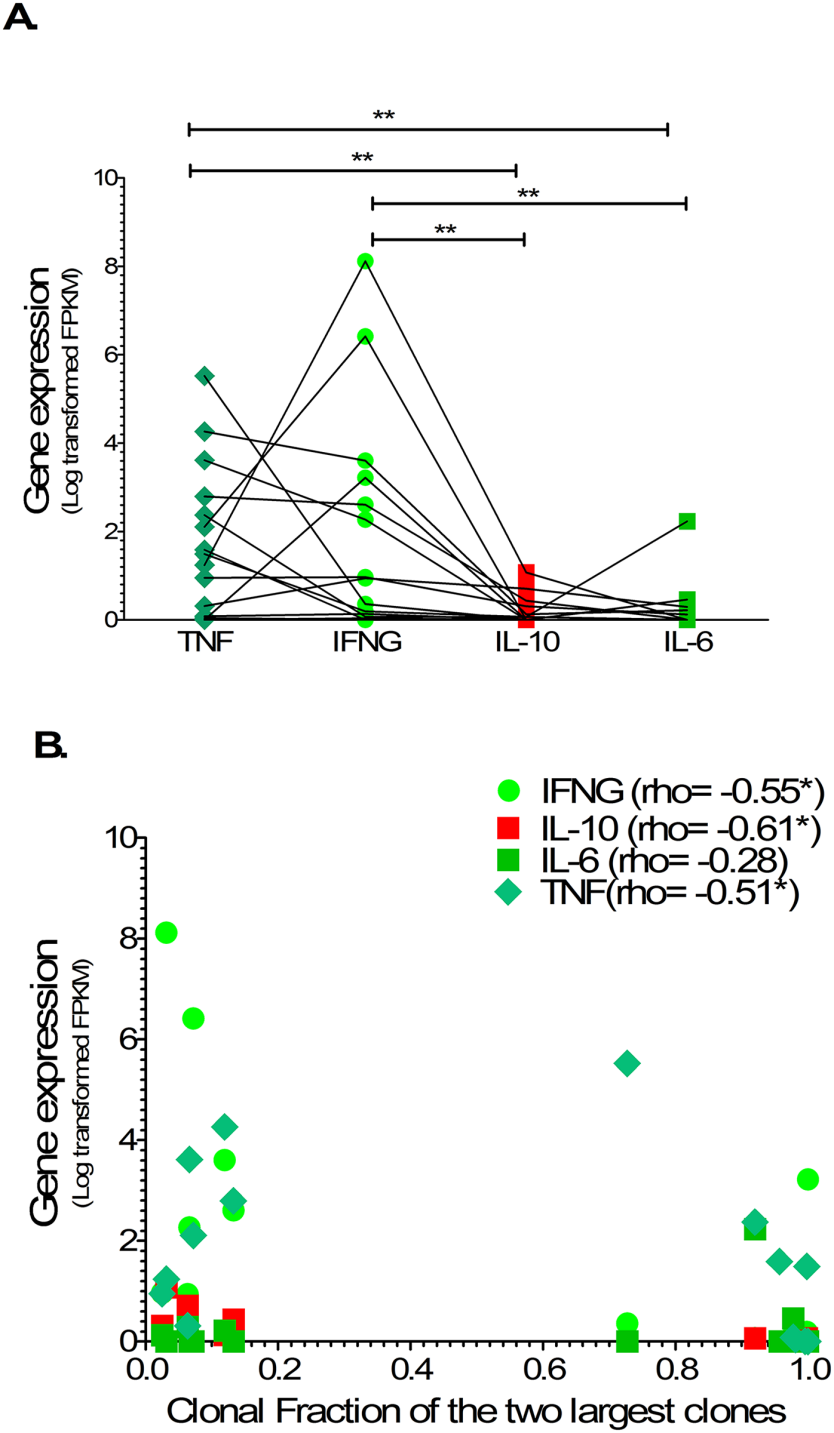
Cytokine mRNA expression profile in non-malignant HTLV-1 infection and ATL. Column plots (A) shows normalized FPKM expression of TNF, IFN, IL-6 and IL-10 linked by patient samples. Statistical analysis: Kruskal-Wallis test with Dunn post-test, 95% confidence interval and Wilcoxon signed rank test. Scatterplot (B) shows correlation between normalized FPKM expression and clonal fraction of two largest clones. Statistical analysis: Spearman correlation. * denotes p<0.05, ** denotes p<0.01, *** denotes p<0.001.

The gene expression of TNFα, IFNγ and IL-10 within ‘ATL-like’ cells was higher in patients with non-malignant infection compared to ATL (p = 0.048, p = 0.031 and = 0.056 respectively) in keeping with relative frequencies of cytokine secreting cells by intracellular cytokine staining. The gene expression of TNFα and IFNγ within ‘ATL-like’ cells was significantly higher than IL-6 (p< 0.01 and p< 0.01 respectively) and IL-10 (p< 0.01 and p< 0.01 respectively) in both patients with non-malignant HTLV-1 infection and ATL as shown in Fig 7A and in keeping with the relative frequencies of cytokine secreting cells by intracellular cytokine staining. The gene expression of TNFα, IFNγ and IL-10 showed significant negative correlation with the clonal fraction of the two largest clones in patients with non-malignant and ATL (p = 0.048, p = 0.031 and = 0.056 respectively) as shown in Fig 7B. Unsupervised hierarchical clustering of all, as well as the differentially expressed, inflammatory transcriptome showed three clusters: a cluster of ATL (seven out of eight patients with ATL), a non-malignant cluster (six out of eight patients with non-malignant) and an overlapping cluster of three patients (one each with indolent ATL, non-malignant with large clones (HKU) and non-malignant without large clones) as shown in S4 Fig.

In summary clonality analysis confirmed that the CD4+CCR4+CD7- cells were derived not only from a single dominant clone (putative ATL cells) but also from multiple non-dominant clones (‘ATL-like’) cells in patients with ATL whilst in non-malignant HTLV-1 infection multiple non-dominant clones contribute to these ‘ATL-like’ cells. A large number of inflammatory transcripts are expressed within CD4+CCR4+CD7- cells in patients with non-malignant HTLV-1 infection and in patients with ATL, of which ten (CCL5, CXCR3, CXCR4, CXCR5, CCR5, CCR6, IFNG, TNF, ICAM1 and IL-RA) were expressed ≥ 2-fold higher in patient with non-malignant compared to ATL. Hierarchical clustering of cytokine expression shows three patterns: ATL, non-ATL and an overlap cluster. TNFα, IFNγ and IL-10 gene expression within CD4+CCR4+CD7- in patients with ATL was lower compared to non-malignant HTLV-1 infection and correlated inversely to size of largest clone.

## Discussion

The study of cellular and plasma immune markers provides an important insight into the pathophysiology of HTLV-1 infection associated clinical states. The differential, network and classification tree analysis of plasma immune markers presented here were in keeping with previously published data. The role of pro-inflammatory cytokines in the pathogenesis of HAM [20, 38–43] was confirmed by higher plasma concentrations of pro-inflammatory cytokines (IFN, IL-17, IL-2 and CXCL10) in patients with HAM.

A positive correlation between plasma concentrations of pro- (TNFα and IL-6) and anti-(IL-10) inflammatory cytokine was present both in patients with non-malignant (AC and patients with HAM) HTLV-1 infection and those with ATL. This correlation has previously been reported in AC and patients with HAM [20] but not ATL and represents the immune consequence of HTLV-1 infection irrespective of clinical state. The pro-inflammatory cytokine concentrations were similar in AC, patients with HAM and patients with ATL but high compared to the published normal range whilst the anti-inflammatory cytokine concentration was higher in patients with HAM or ATL compared to AC. Patients with aggressive ATL had higher plasma pro- and anti-inflammatory cytokines concentrations compared to patients with indolent ATL. This is in keeping with the results of a Japanese study which found high plasma IL-6 and IL-10 concentrations (anti-inflammatory cytokines) in patients with aggressive ATL compared to ACs and patients with indolent ATL[12] and a Middle-Eastern study which showed high plasma IL-10 concentrations in patients with ATL[13]. A recent Brazilian study also showed raised plasma IL-10 in patients with HAM compared to AC [20]. These data confirmed not only that ATL is associated with high anti-inflammatory plasma cytokine concentrations, especially in aggressive disease, but also that this is seen in patients with HAM. Importantly the plasma cytokine profile is established early and rather than immediately preceding or predicting aggressive ATL.

In the UK cohort, Incident ATL has occurred predominantly amongst patients with HAM [44]. Similarly, a South American study highlighted the high co-incidence of HAM and ATL [45]. The higher plasma IL-10 which positively correlated with IFNγ concentrations was present in both patients either with ATL or with HAM. The role of these interactions in progression to ATL merit further investigation.

Pro and anti-inflammatory cytokines are secreted by a variety of cells including macrophages, monocytes, lymphocytes and non-haematopoietic cells. The overwhelming majority of HTLV-1 infection burden is within CD4+T cells in both non-malignant and ATL infection [6, 23, 24, 35]. The cytokine producing capacity of monocytes and lymphocytes might help elucidate the direct contribution of HTLV-1 infected, ATL and micro-environment cells to the plasma cytokine profile. The cytokine producing capacity of CD4+T cells has been shown to contribute to the plasma cytokine profile in ATL[13, 15]. The absolute frequency of IL-10 secreting CD4+T cells in patients with ATL was higher compared to AC and HAM. CD4+T cells are a mixture of infected and uninfected cells in patients with HTLV-1 infection.

In ATL the majority of infected cells are derived from a single dominant leukemic clone amongst thousands of non-dominant clones whilst thousands of non-dominant clones of varying size contribute to the total infection burden in non-ATL HTLV-1 infection [29, 36, 37, 46]. CD4+CCR4+CD26-CD7- cells have been shown to harbour the dominant clone in ATL and these cells are also present in non-malignant HTLV-1 infection[23, 27, 47, 48]. We have demonstrated that these cells are made up of hundreds of non-dominant clones (‘ATL-like’ cells) in patients with non-malignant HTLV-1 infection and a single dominant clone (putative ATL cells) amongst tens of smaller non-dominant clones (‘ATL-like’ cells) in patients with ATL. This is different to previous work, which showed infected cells with an ‘ATL-like’ immunophenotype harbour only dominant clones, is due to the higher sensitivity and quantification of our clonality techniques (LMPCR-HTS and TCR sequencing compared to inverse PCR).

The cellular cytokine producing profile, inflammatory transcriptome and clonality within ATL and ‘ATL-like’ cells was directly studied for the first time. CD4+CCR4+CD7- cells had pro- and anti-inflammatory cytokines producing capacity at protein (intracellular cytokine staining) and mRNA (RNA sequencing) level in non-malignant HTLV-1 infection and in ATL. The CD4+CCR4+CD7- cytokine producing capacity of not only anti- but also pro-inflammatory cytokines was higher in patients with ATL compared to AC and patient with HAM. These findings appear to be contrary to the assumption that these cells produce only anti-inflammatory cytokine based on the extrapolation of the high plasma IL-10 concentration and high frequencies of IL-10-producing CD4+cells in patients with ATL findings. However, the cytokine producing capability of CD4+CCR4+CD7- cells in patients with ATL, measured by both ICS and RNA sequencing, was extremely low raising the suspicion the cytokine producing cells might not be the putative ATL cells but the ‘ATL-like’ cells. This was supported by the clonality analysis. The size of the dominant clone by LMPCR-HTS and TCR clonality correlated inversely with the frequency of cells secreting any cytokines by ICS and cytokine mRNA expression. Very few studies have been performed to study directly the cytokine producing capability of ATL and ‘ATL-like’ infected cells. CD4+CCR4+CD25+ (HTLV-1 infected) cells have been showed to be capable of secreting IFNγ in patients with HAM (21, 22, 40]. Only a minority of CD4+CD25+ cells in patients with ATL have been shown to be capable of cytokine secretion [49]. The cause and implication of the raised cytokine producing ‘ATL-like’ cells of non-dominant clones in patients with ATL is not completely clear and needs further investigation.

The higher anti- and lower pro- inflammatory cytokine producing capability of ‘ATL-like’ infected cells compared to HTLV-1 uninfected CD4+ T cells was demonstrated in patients with non-malignant HTLV-1 infection. This finding could be due to expansion of rare CD4+T-cell subsets or a change in the differentiation of host cells by HTLV-1 infection. CD4+CCR4+ and CD4+CD7- T cells have been shown to have Th2 and regulatory T cells cytokine skewing in healthy individuals [50–52]. These suggest HTLV-1 infection might select the differentiation and immune function of infected cells.

The main limitation of the current study is that the experiments were performed only on PBMCs and thus the cytokine profile of non-circulating lymphocytes in lymph nodes and other tissues remains to be elucidated. Secondly the evidence for cytokine producing capability of dominant and non-dominant clone(s) in patient with ATL is indirect. Direct evidence would require unusual volumes of patient blood samples as well as technical advances (improving purity and yield of sorting techniques). Hence, the indirect correlation data of cytokine producing capacity by ICS and clonality by LMPCR-HTS was confirmed by an independent technique (RNA sequencing to study inflammatory transcriptome and TCR clonality) in the same and in additional patient samples.

### Conclusion

There is an absolute increase in ‘ATL-like’ cells made up of non-dominant infected clones in patients with non-malignant HTLV-1 infection (AC and patients with HAM). There is a further expansion of these ‘ATL-like’ cells in ATL along with the presence of ATL cells. The ‘ATL-like’ cells from non-dominant clones have a distinct cytokine producing pattern and contribute directly to the plasma cytokine profile in both non-malignant HTLV-1 infection and possibly in ATL. The ‘ATL-like’ cells of the dominant clone (the putative ATL cells) possess little or no cytokines producing capability. There was progressive loss of pro-inflammatory cytokine producing capacity from non-ATL (uninfected) through ‘ATL-like’ (infected) to ATL (malignant) cells and we hypothesise that this represents stages in the transformation process.

## Supporting information

S1 Table Demographic data on study patients with non-malignant HTLV-1 infection and ATL. (XLSX)

S2 Table Sequence of primer used for LMPCR-HTS. (DOCX)

S3 Table Differential inflammatory transcriptome in patient with non-malignant HTLV-1 infection and ATL. (XLSX)

S1 Fig Plasma cytokine concentrations in ATL. A-F) Aligned column plots of plasma cytokine/chemokine concentrations in indolent and aggressive ATL. The bar represents median values. Statistical analysis: Kruskal-Wallis test with Dunn post-test, 95% confidence interval and Wilcoxon signed rank test. * denotes p<0.05, ** denotes p<0.01, *** denotes p<0.001.
(TIF)

S2 Fig Column 1 shows gating strategy for CD4+T cells as well as CCR4+CD7- and non CCR4+CD7- subsets. The histogram shows the expression in count of cytokine staining cells in representative patient with asymptomatic carriers (orange), HTLV-1 associated myelopathy (blue) and adult T-cell leukaemia lymphoma (red).
(TIF)

S3 Fig CD14+ monocytes (A-C) and CD8+ T-cells (D-G) cytokine producing profile in non-ATL HTLV-1 infection and ATL. Bar column plots showing relative frequency of CD8+ T cells and CD14+ monocytes in asymptomatic carriers (AC), patients with HTLV-1 associated myelopathy (HAM) and adult T-cell leukaemia/lymphoma (ATL). The bar represents mean values and error bar the standard deviation. Statistical analysis: Kruskal-Wallis test with Dunn post-test, 95% confidence interval and Wilcoxon signed rank test. * denotes p<0.05, ** denotes p<0.01, *** denotes p<0.001.
(TIF)

S4 Fig Hierarchical clustering of inflammatory transcriptome in patients with non-malignant HTLV-1 infection and ATL. Heatmap of all (A) and significantly differential (B) expressed inflammatory transcriptome shows clustering of patient with ATL, non-malignant and overlap.
(TIF)
