# Supplementary material for: Switching and loss of cellular cytokine producing capacity characterize in vivo viral infection and malignant transformation in human T- lymphotropic virus type 1 infection
Source: PLoS Pathog. 2018 Feb 14;14(2):e1006861. doi: 10.1371/journal.ppat.1006861 (PMC5828519; doi:10.1371/journal.ppat.1006861)
Supplement: S2 Table — (DOCX) [file ppat.1006861.s002.docx]

| Primer | Label | Sequence (5’ to 3’) |
| --- | --- | --- |
| Nested PCR1 Forward primer | BIO3 | CCTTTCATTCACGACTGACTGCCG |
| Nested PCR1 Reverse primer | BIO4 | TCATGATCAATGGGACGATCA |
| Nested PCR1 Forward primer | P5-Bio5-**s***** | AATGATACGGCGACCACCGAGATCTACACNNNNNNNNTCGTCGGCAGCGTCAGATGTGTATAAGAGACAGNNNNNTGGCTCGGAGCCAGCGACAGCCCAT |
| Nested PCR2 Reverse primer | P7 | CAAGCAGAAGACGGCATACGAGAT |
| DNA adaptor  Long arm | Vu-Long-N7**-TAG  (Bio4-P7- N7**-SeqPrimer-TAG-) | TCATGATCAATGGGACGATCACAAGCAGAAGACGGCATACGAGATXXXXXXXXGTCTCGTGGGCTCGGAGATGTGTATAAGAGACAGNNNNNNNNCAGAGATCAATAGT |
| DNA adaptor  Short arm | VU_SHORT_TAG | p-CTATTGATCTCTGAAAAAAAAAAAAA |

Supplementary Table 2: Sequence of primers used for LMPCR-HTS.
