# Supplementary figures and images for: Switching and loss of cellular cytokine producing capacity characterize in vivo viral infection and malignant transformation in human T- lymphotropic virus type 1 infection
Source: PLoS Pathog. 2018 Feb 14;14(2):e1006861. doi: 10.1371/journal.ppat.1006861 (PMC5828519; doi:10.1371/journal.ppat.1006861)

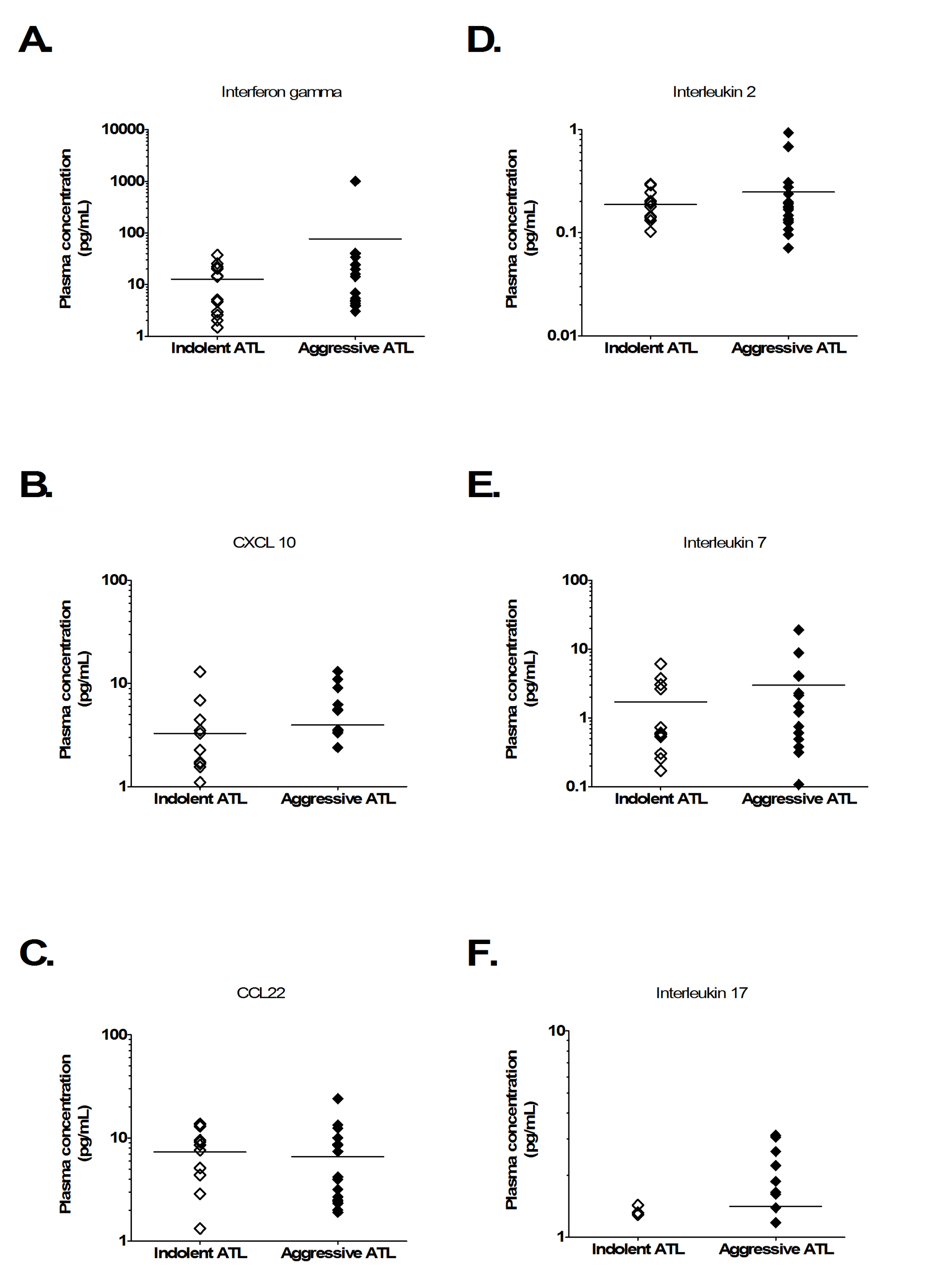

Supplement: S1 Fig — A-F) Aligned column plots of plasma cytokine/chemokine concentrations in indolent and aggressive ATL. The bar represents median values. Statistical analysis: Kruskal-Wallis test with Dunn post-test, 95% confidence interval and Wilcoxon signed rank test. * denotes p<0.05, ** denotes p<0.01, *** denotes p<0.001. (TIF) [file ppat.1006861.s004.tif]

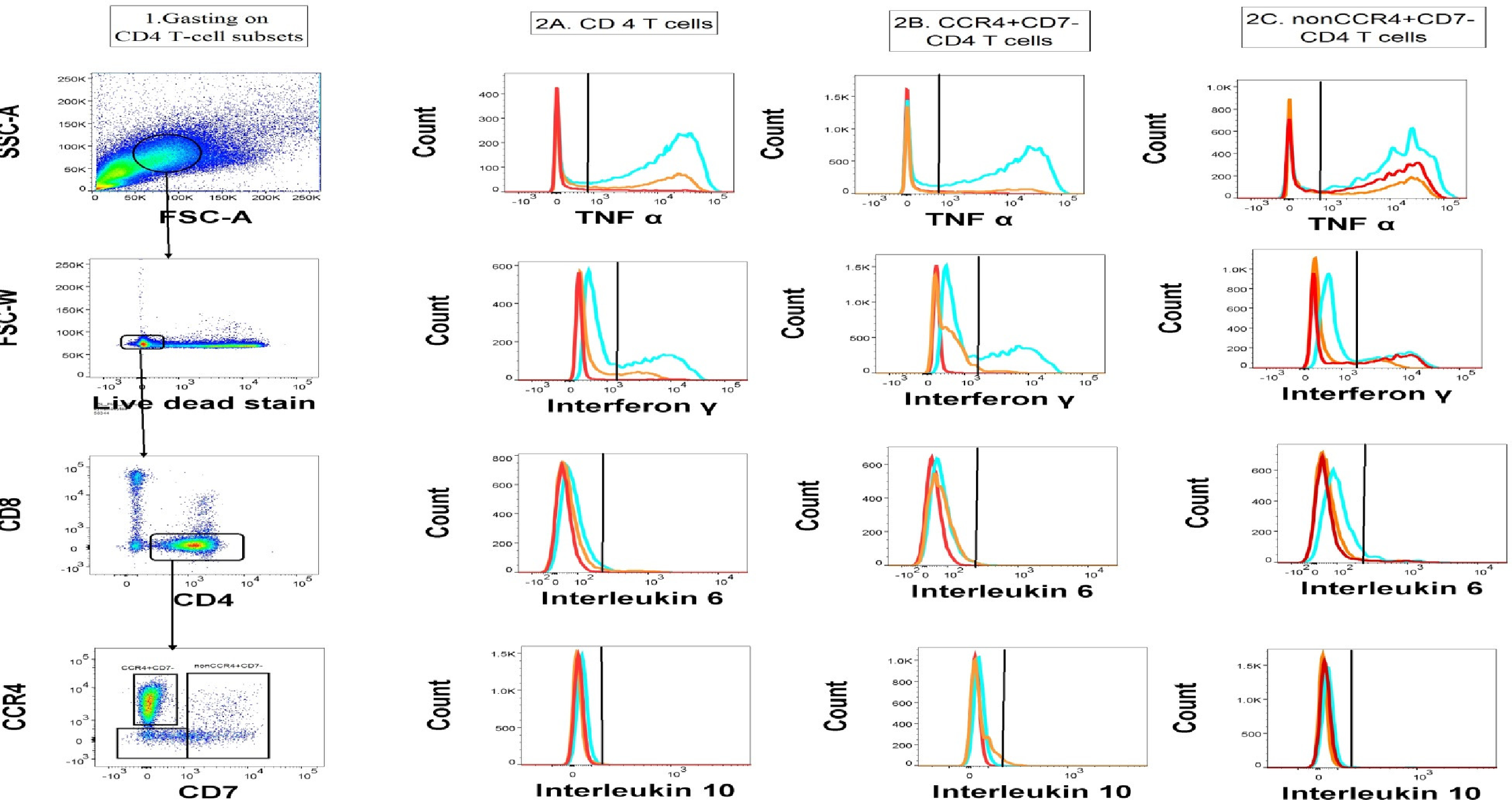

Supplement: S2 Fig — The histogram shows the expression in count of cytokine staining cells in representative patient with asymptomatic carriers (orange), HTLV-1 associated myelopathy (blue) and adult T-cell leukaemia lymphoma (red). (TIF) [file ppat.1006861.s005.tif]

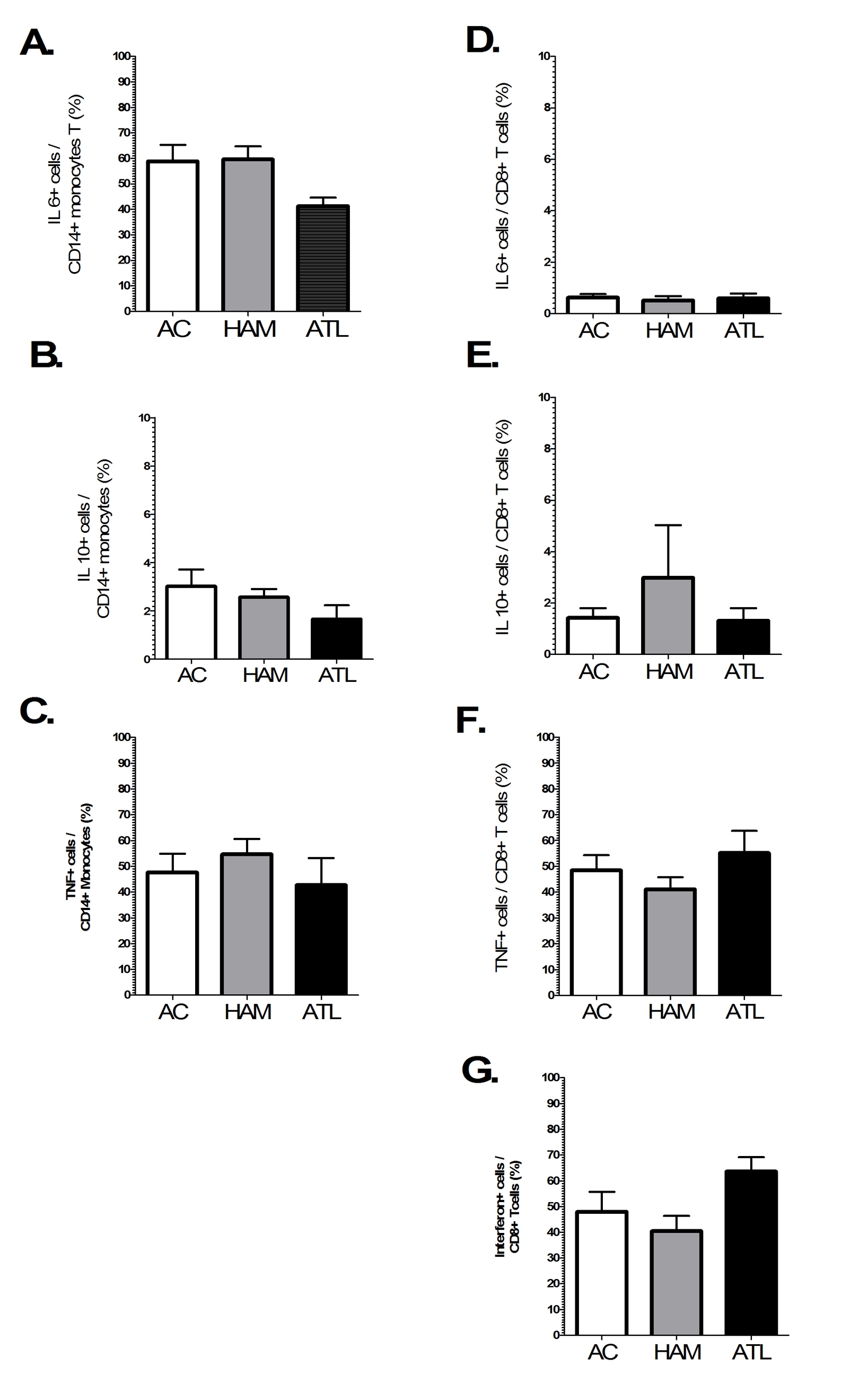

Supplement: S3 Fig — Bar column plots showing relative frequency of CD8+ T cells and CD14+ monocytes in asymptomatic carriers (AC), patients with HTLV-1 associated myelopathy (HAM) and adult T-cell leukaemia/lymphoma (ATL). The bar represents mean values and error bar the standard deviation. Statistical analysis: Kruskal-Wallis test with Dunn post-test, 95% confidence interval and Wilcoxon signed rank test. * denotes p<0.05, ** denotes p<0.01, *** denotes p<0.001. (TIF) [file ppat.1006861.s006.tif]

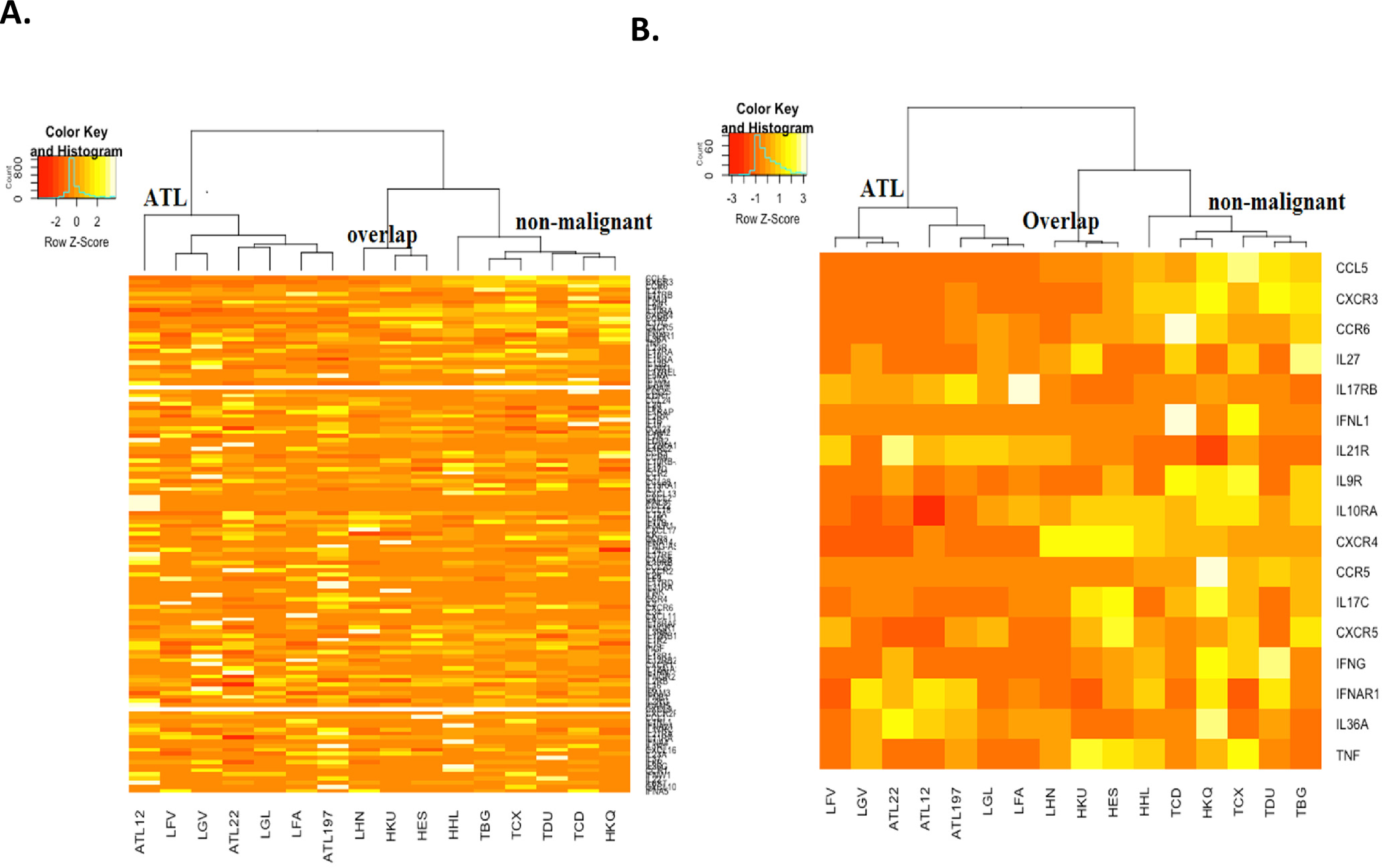

Supplement: S4 Fig — Heatmap of all (A) and significantly differential (B) expressed inflammatory transcriptome shows clustering of patient with ATL, non-malignant and overlap. (TIF) [file ppat.1006861.s007.tif]
